# A Lightweight Prototype of a Magnetometric System for Unmanned Aerial Vehicles

**DOI:** 10.3390/s21144691

**Published:** 2021-07-09

**Authors:** Antonino Pisciotta, Giovanni Vitale, Salvatore Scudero, Raffaele Martorana, Patrizia Capizzi, Antonino D’Alessandro

**Affiliations:** 1 Istituto Nazionale di Geofisica e Vulcanologia, Sezione di Palermo, 90146 Palermo, Italy; fabio.pisciotta@ingv.it; 2 Istituto Nazionale di Geofisica e Vulcanologia, Osservatorio Nazionale Terremoti, 00143 Rome, Italy; giovanni.vitale@ingv.it (G.V.); salvatore.scudero@ingv.it (S.S.); antonino.dalessandro@ingv.it (A.D.); 3 Department of Earth and Marine Sciences (DISTEM), Università degli Studi di Palermo, 90123 Palermo, Italy; patrizia.capizzi@unipa.it

**Keywords:** magnetometry, UAV, archaeology, airborne magnetometer, controlling unit

## Abstract

Detection of the Earth’s magnetic field anomalies is the basis of many types of studies in the field of earth sciences and archaeology. These surveys require different ways to carry out the measures but they have in common that they can be very tiring or expensive. There are now several lightweight commercially available magnetic sensors that allow light-UAVs to be equipped to perform airborne measurements for a wide range of scenarios. In this work, the realization and functioning of an airborne magnetometer prototype were presented and discussed. Tests and measures for the validation of the experimental setup for some applications were reported. The flight sessions, appropriately programmed for different types of measurements, made it possible to evaluate the performance of this detection methodology, highlighting the advantages and drawbacks or limitations and future developments. From the results obtained it was possible to verify that the measurement system is capable of carrying out local and potentially archaeological magnetometric measurements with the necessary precautions.

## 1. Introduction

Magnetometry is the branch of geophysics which deals with the Earth’s magnetic field and its spatio-temporal variations. The variations in the Earth’s magnetic field may occur at different scales (i.e., global, regional, and local) and may be ascribable to different sources, either natural or anthropogenic [1]. The study of magnetic anomalies has several applications due to its passive, fast, and noninvasive detection capability [2,3] such as: rock types mapping, mineral exploration [4], reconstruction of the geometry of geological bodies [5], detection of buried objects at different depths, determination of heavy metal pollution in the soil, and archaeological investigations [6,7,8]. Each application requires a proper type of magnetic sensor(s) and a targeted surveying method that mainly depend on the scale of the anomalies to investigate and on the desired resolution.

Satellite measurements are used for global magnetic models with 1–5-km resolution [9]. Airborne or shipborne surveys investigate the regional anomalies with resolution in the order of hundreds to thousands of meters [10]. Ground-based magnetic measurements investigate the shallower ground reaching sub-metric resolution.

In recent years, archaeological prospecting has benefited from important improvements due to the availability of high-resolution remote sensing techniques and their integration with geophysical methods with multiscale approaches [11]. This can provide a valid help in geoarchaeological contexts, with multiple purposes such as the historical reconstruction of landscapes and sites [12,13], preventive archaeology [14], management and conservation of the archaeological heritage [15,16], and non-invasive diagnosis through microgeophysical techniques [17,18].

Among the geophysical methods, magnetometry has proved very effective for archaeological purposes, due to its ability in detecting buried anthropic structures as these features, generally, have different magnetic properties from the cover terrain [19,20,21,22,23,24].

Recently, the extensive development of unmanned aerial vehicles (UAVs) enabled to partly fill the large gap between airborne and ground-based magnetic surveys. Therefore, UAV platforms equipped with appropriate magnetic sensors, allow to perform faster, more reliable, and even safer surveys [25,26]. In fact, they overcome the main limitations of the traditional ground-based ones (difficult to access, uneven terrains, presence of obstacles) and the costs of airborne surveys. Several prototypes have been developed in recent years, adopting different technical choices about the type of UAV and the type of magnetic sensor [27,28,29,30,31,32].

Significant progress has been achieved in the integration and testing of the UAV-magnetometer system to date. The UAV-magnetometer system outperforms portable and aeromagnetic equipment, whether it is based on a fixed-wing [33,34] or multi-rotor UAV [28,35,36], a single magnetometer [28,33] or many magnetometers [34]. However, due to the low quality of magnetic data and a lack of interpretation methods for UAV-based magnetic surveys, research on the processing and interpretation of UAV magnetic data is relatively rare and difficult [37].

Recent studies have been done to evaluate the feasibility of using UAVs equipped with magnetometric systems for archaeological research. For this issue, however, lightweight systems are required and, at the same time are capable of performing measurements with high spatial resolution and high sensitivity, flying at low altitude and with low energy consumption. A comparative analysis was made by [38] to test the impact of different kinds of UAVs on magnetometers in order to determine the most optimal distance of the sensors. A system with a microwire magnetometer on UAVs was developed by [39] and tested on archaeological features. The noise caused by an 8-rotor copter equipped with a cesium-vapor magnetometer was analyzed and filtered by [31] on data acquired in an archaeological site. The use of a fluxgate three-component magnetometer was proposed by [40], exploiting its lightness and its low power consumption, mounting it on a girocopter and on an aircraft and testing the systems on different sites, including archaeological ones. The problems encountered so far concern the low resolution, the noise generated by the rotors and the high energy required to drive non-light UAVs.

This paper presents the realization of a lean acquisition system for a high-resolution 3-axial fluxgate magnetometer integrated into a lightweight UAV platform. The system georeferences data by means of a GPS, stores data on SD memory, and transmits data via a radio module. We evaluated the performance of the device by means of laboratory tests and of an archaeological site. Results indicate that it is a reliable system capable of performing magnetic surveys for different applications.

## 2. Materials and Methods

### 2.1. Design Specification

**Figure 1:**
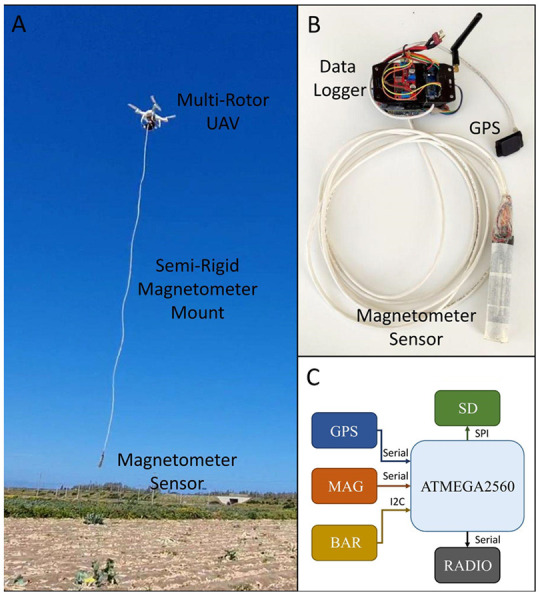
The unmanned aerial vehicle (UAV)-magnetometer system. (**A**) The UAV equipped with the data logger carrying the magnetometer sensor; (**B**) the magnetometer system, data logger, and sensors; (**C**) diagram of the data acquisition and control system.

The device consists of a magnetic sensor and a controlling unit which has been appropriately designed to be integrated into UAV platforms (Figure 1). The design followed the criteria to minimize dimensions and weight in order to achieve better performance with the most popular UAV platforms whose payload is usually limited (few hundreds of g). Another design criterion was to minimize power consumption. The power to the system is directly supplied by the UAV platform and, for this reason, it is important to save power to ensure longer surveying flight times. Finally, the design paid attention to avoid electro-magnetic interference between the UAV platform, the controlling unit, and the sensing part. Details about the various components are provided in the following paragraphs.

The UAV platform employed is a DJI Phantom 4 model. It provides the power to the device, but its voltage output is different from the voltage required by the controlling unit (5 V) and for the sensing unit (9 V). For this reason, the supply is obtained by means of two step-down modules that dissipate little energy and save the UAVs autonomy. These power converters can cause problems of interference due to switching operations. However, the devices used have an internal linear voltage stabilizer dc-dc converter, which allows using a step-down converter without additional filters.

The controlling unit is based on an Atmega 2560 microcontroller (Atmel Corporation Technology, San Jose, CA, USA). It manages all the peripherals, performs calculations, and logical operations. The external peripherals implemented in the controlling unit are: GPS, storage memory, radio transmission, barometer, and, of course, the magnetic sensor (Figure 1C).

The GPS is an essential system: It provides the exact timing and location (geodetic longitude, latitude, and altitude) for each magnetic measure. This is a supplementary system, since the UAV exploits its own embedded GPS for the navigation. The use of an independent GPS allowed to develop an independent acquisition system, not tied to a single UAV system, but usable also for other light UAVs with similar characteristics. The orientation is settled in the planning phase and kept by the UAV IMU (inertial measurement unit) sensors (Shenzhen DJI Sciences and Technologies Ltd., Shenzhen, China).

The geodetic altitude can be affected by significant uncertainties. A barometer was integrated into the device so that the true altitude could be estimated with a greater confidence. Barometer data are filtered with a moving average with a window of ten samples. A graphical interface shows and plots in real time the recorded data into a personal computer, by means of a serial transceiver. This system also allows telemetry and enables switching among the operating modes. All the above-mentioned peripherals, together with the magnetic sensor, are controlled via a serial connection UART adapter. The serial port manages the communication between the microcontroller and the SD module. Data are then stored in textual format on a removable SD card.

The sensing unit is a commercial 3-axial fluxgate magnetometer (model 1540 by Applied Physics System, Mountain View, CA, USA). The sensor has dynamic range of ±0.65 Gauss and a resolution of 0.001 mG. A fluxgate magnetometer consists of a small, magnetically sensitive core wound by two coils of wire. An alternating electric current passes through the primary coil, bringing an air gap into alternated magnetic saturation. The induced current on the secondary coil is then measured. In the absence of magnetic fields, the current on the primary and secondary winding are balanced, but when the sensor is exposed to an external magnetic field, the air gap enters saturation asymmetrically. Thus, the alternating magnetic field and the induced output current are out of step with the input current; this phase shift depends on the intensity of the magnetic field. The output current is proportional to the magnetic field that strikes the air gap. Because they are rugged and lightweight, the fluxgate sensors are the most widely adopted in these portable systems [28,32,33,35,37,41].

While the controlling unit is in-built with the frame of the UAV platform, the magnetometer is freely suspended in vertical position to a 4-m long wire below the UAV. We chose a semi-rigid magnetometer mount because it is the better solution of safety and low payload. In fact, the payload package, including magnetometer, data storage, and communications module, has a weight of 385 g. In this way the UAV’s total payload is of 1773 g. However, this configuration presents both pros and cons. If from one hand, it excludes any possible magnetic interference due to the rotors or to the circuits, on the other, it introduces some noise in the measures due to oscillations of the sensor. This solution, which avoids dealing with magnetic interference, has been adopted also in other analogous experimental magnetic systems [36,41,42,43].

The magnetic field induced by the UAV and the acquisition unit is not easy to assess and requires complex compensation algorithms to be filtered out [34,44,45,46,47,48]. Conversely, in the pendulum-like arrangement, the characteristics of the oscillating system being fixed (length of the wire and mass of the sensor), the period of oscillation is constant and can be easily recognized and filtered out. Moreover, some precautions during the flight can limit this effect, such as avoiding sudden changes in velocity or direction. The oscillation of the magnetometer is almost null at constant cruising, as will be later discussed in the paragraph devoted to the data analysis.

The three components of the magnetic field are measured separately, and the microcontroller calculates the total magnetic field in real-time. This operation introduces negligible offset (−0.0018 nT) in the values of the total magnetic field [49]. The sampling frequency of the magnetometer is set to 5 Hz and the unit of measurement is milligauss (mG).

Another critical point concerns the flight autonomy. The battery capacity is 5870 mAh. We found that this allows about 13 min of flight with the above discussed payload, with a flight speed of 1.8 km/h. In practice this allows, for example, a survey area of about 400 m^2^ with parallel sweep lines 2-m spaced to cover.

For more technical details, the operating modes of the device, and the first experimental tests to validate the performance of the prototype, which include a comparison with an overhauser magnetometer (GEM-19 from GEM Systems), please refer to [29,49,50].

### 2.2. UAV Survey

The device was tested in an archaeological site where part of the features has been excavated and brought to light, while part of the remains is still buried in the shallow subsurface. The site is the Himera archaeological park which is located in Sicily (Italy) and represents the rest of the ancient Greek city called Himera [51]. This town was founded near the mouth of the Northern Imera River in 648 BC. The settlements are distributed in two distinct areas: the lower town near the mouth of the river and the higher town, on the southern hill. In this archaeological site there are few geophysical studies carried out in the past [52,53]. The higher town has already been extensively excavated and many archaeological remains have been brought to light. We chose our test sites in the higher town. In this area we estimated that the difference in susceptibility between the calcarenite limestone (4–5) of the archaeological remains and the clayey covering soil (about 24, see [53]) is sufficiently high to generate clearly detectable magnetic anomalies.

**Figure 2:**
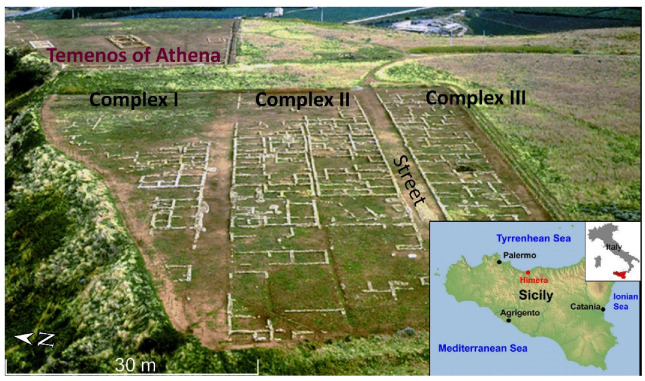
Aerial view (from west) of the test site of Himera and its geographical location.

**Figure 3:**
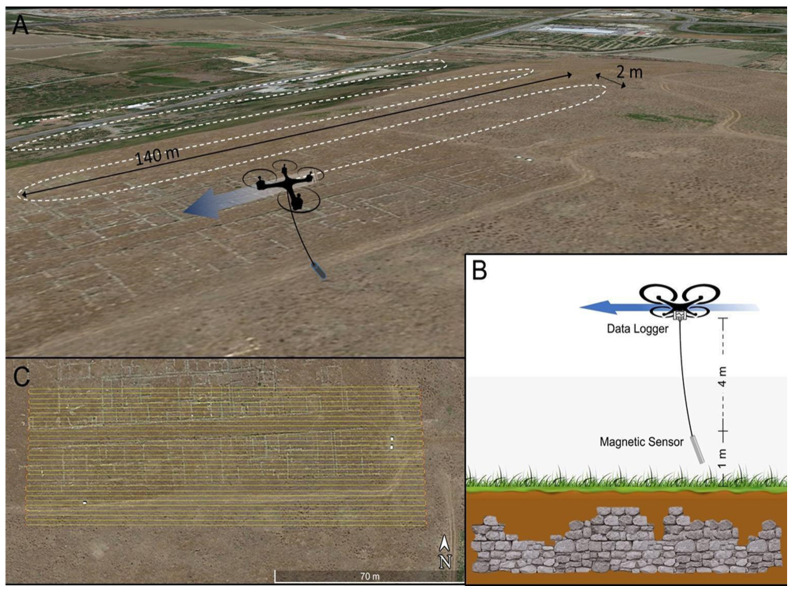
(**A**) UAV flight plan over the test site. (**B**) Scheme of the acquisition of the UAV-magnetometer platform. (**C**) The path of the performed mission follows E-W oriented tracks spaced 2 m.

Two areas were investigated. The first survey was performed following E-W oriented tracks, the second one follows N-S tracks. The first area partially covers three complexes of houses, previously unearthed, west of Athena’s Temenos. Here the archaeological features are arranged to form an orthogonal pattern with the main features (e.g., roads, foundations, walls) oriented ~ENE-WSW and the secondary one ~NNW-SSE (Figure 2). The flight plan partially covered two unearthed house complexes, to test the system on known results, and laterally extended in unexcavated land, to find new results (Figure 3c).

A second survey was performed in an unexcavated area immediately south of the previous one, in which it is assumed that the urban plant continues. In this area we expected to find anomalies caused by other archaeological complexes of the same periods and, consequently, with characteristics similar to those excavated, but also structures dating back to a previous settlement.

Hereafter we describe the set-up of the test. During mission planning, the routes were settled to keep a rectangular grid using a back-and-forth pattern without rotation. The velocity at which the UAV performed the magnetic survey was a tradeoff between the batteries’ availability, the size of the area to investigate, and the desired resolution. The survey velocity adopted in the performed tests was equal to 2.0 m/s and resulted in an averaging spacing of about 0.4 m between two consecutive samples. The inter-distance should be calibrated to be comparable with the dimension of the features to detect. The spacing between two adjacent and opposite tracks should be adequately dimensioned. In this case, it is equal to 2 m and it takes into account that most target features are elongated objects (foundations, walls, or roads) rather than punctual features (Figure 3). Having considered a not-too-small spacing between the profiles, together with taking measurements in one direction, is a good compromise, at this stage, between the speed of execution, linked to the UAV’s autonomy, and the detectability of the main structures when these are elongated in the direction transverse to that of flight. The noisy portions of the signal in correspondence of the turn-round points have been removed for a length of about 10 m, in order not to consider the parts of the flight paths in which the speed was not nearly constant and, consequently, to exclude the measurements affected by abnormal sensor oscillations (see Section 3.1). For this reason, the UAV coverage path planning is greater than the magnetometric map layout. The minimum flight elevation has been set to +6 m (+7 m for the second survey) with respect to the basepoint of the survey and, considering the length of the wire connecting the UAV to the sensor, the latter travels at about 2 m (+3 m) above the ground level. The different flight elevations were due to tall grass in the second area which prevented a lower flight elevation from being set. The UAV travels at constant absolute elevation, while the distance from the ground may vary because the terrain surface is not perfectly flat. In order to perform gradiometric measures, we repeated both surveys at greater elevation (+1 m). This value corresponded to the minimum vertical step allowed by the controlling software of the UAV which did not allow a finer control in the vertical position. Gradiometry has several pros: The measure was independent from temporal variations; and it was able to somehow isolate the signals coming from shallow sources (i.e., archaeological features), resulting in less sensitive to deeper, larger scale, sources [24].

## 3. Results

### 3.1. Data Analysis

**Figure 4:**
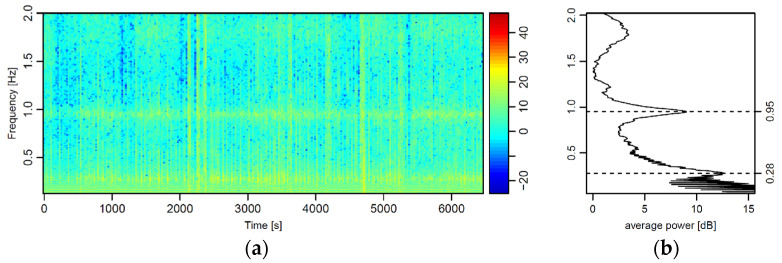
Spectrogram (in the band 0.125–2 Hz) of the magnetic signal acquired during various flights in the test site (**a**) and relative average power (**b**). The two peaks at 0.28 and 0.95 Hz are the effects of the oscillation of the sensor suspended underneath the UAV platform.

The choice to suspend the magnetic sensor below the UAV by means of a wire gave rise to harmonic oscillations of the sensor. Such oscillations continuously affected the magnetic signal recorded during the field test. The spectrogram (Figure 4) clearly highlighted two main frequencies: one at 0.28 Hz (3.57 s) characterized with a greater power, and one at 0.95 Hz (1.05 s) with minor power. We verified that during hovering maneuvers, the oscillation at 0.28 Hz rapidly disappears. Nevertheless, the secondary oscillation at 0.95 Hz keeps affecting the signal. For this reason, the peak at 0.28 Hz was the one ascribable to the primary pendulum motion of the sensor. The peak at 0.95 Hz was interpreted as the effect of the feedback of the UAV, which continuously corrected its spatial position, as transmitted to the sensor via the non-rigid cable.

Basically, the first and last meters of each profile were affected by the acceleration/deceleration and change of direction of the UAV which generated large oscillations of the sensor. Once the planned flight speed was reached, it remained almost constant, and this ensures that the oscillations of the sensor are quite regular. For this reason, it is advisable to plan a larger UAV coverage of the area to be investigated and cut every beginning and end of the profiles.

**Figure 5:**
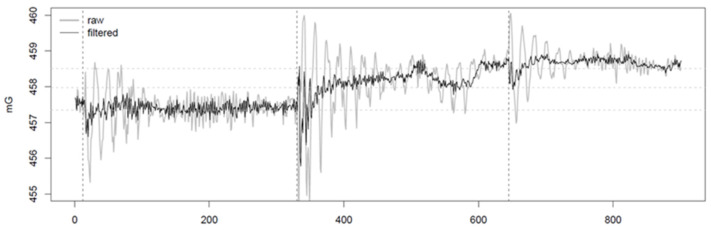
Example of filtering of the magnetic signal by means of the stop-band filters on a portion of 900 samples (3 min) which includes three adjacent magnetic sections. The vertical lines indicate the turn-round positions, immediately before the maximum amplitude of the 0.28 Hz oscillations. The horizontal lines (mean value for each section) highlight a horizontal (southwards) gradient of about 0.6 mG between two consecutive sections.

The narrow bands corresponding to the two frequency peaks can be easily removed with the application of targeted stop-band filters (Figure 5). Such inconvenience in the magnetic signal is far more easily solvable with respect to the electromagnetic interferences potentially triggered with the sensor placed in the proximity of the UAV.

**Figure 6:**
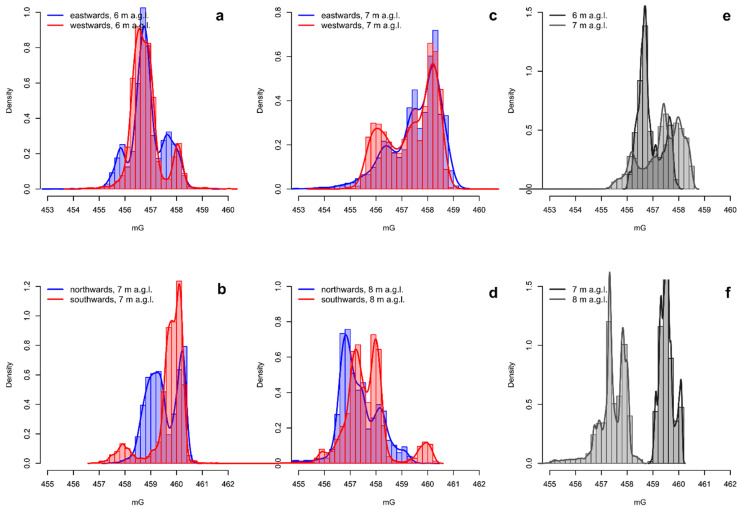
Comparison of frequency distributions (histograms and kernel density plots) between the opposite surveying directions acquired at different elevations above ground level (**a**–**d**, respectively) and at the two corresponding elevations after the spatial interpolation (**e**,**f**).

The fluxgate magnetometers are directional sensors; this means that the measure is somehow dependent on the orientation of the sensor. To avoid this effect, the surveys have been carried out following opposite tracks using a back-and-forth pattern without rotating the UAV. Despite this, a kind of directional dependence still occurs because it is not possible to maintain the direction constant, both because of the small lateral deviations, essentially caused by the wind, and by the inertia of the sensor attached to the cable, which causes opposite slopes along parallel tracks. In order to assess the degree of directional dependence, we analyzed the frequency distributions of the measures acquired during the tests after being filtered for the oscillations. In Figure 6, we compared the frequency distributions of the magnetic data acquired following the opposite surveying directions after being filtered for the harmonic oscillations. The general trends of the pairs of curves were coherent. Generally, eastwards and westwards tracks were more coherent with respect to northwards and southwards tracks. The main peaks were centered at the same values, even though there were misalignments ranging from ~0.2 to ~0.6 mG. Greater differences may arise in the secondary peaks characterized by lower and higher magnetic values at both elevations. We suggest that the directional effect may be negligible for the background magnetic field, while it may be somehow more relevant for local anomalies. This effect can be easily corrected by averaging the magnetic values between two adjacent (and opposite) survey tracks [6]. However, it may not be necessary in case of production of 2D magnetic maps. In the presented case study, the data were interpolated by kriging to produce two maps of the total magnetic field, one for each survey altitude. For the interpolation, the kriging method was used by calculating the variogram for each set of measures, in order to consider the anisotropy inherent in the large difference between the sampling along the overflight direction and that along the direction perpendicular to it, looking at the same time to maintain the resolution of the survey. The variogram model mathematically specifies the spatial variability of the data set and the resulting grid file. The variogram models applied on data are of the gaussian type. We considered a maximum range of 4 m and a nugget effect based on an estimated error variance of about 15 cm. These parameters were chosen by searching the best fit between the theoretical and experimental variogram points. The interpolation weights, which are applied to data points during the grid node calculations, are direct functions of the variogram model. The spatial interpolation enhances the peaks, removes the codas, and also indirectly reduces the directional effects without any ad-hoc correction (Figure 6e,f).

### 3.2. Magnetic Maps

**Figure 7:**
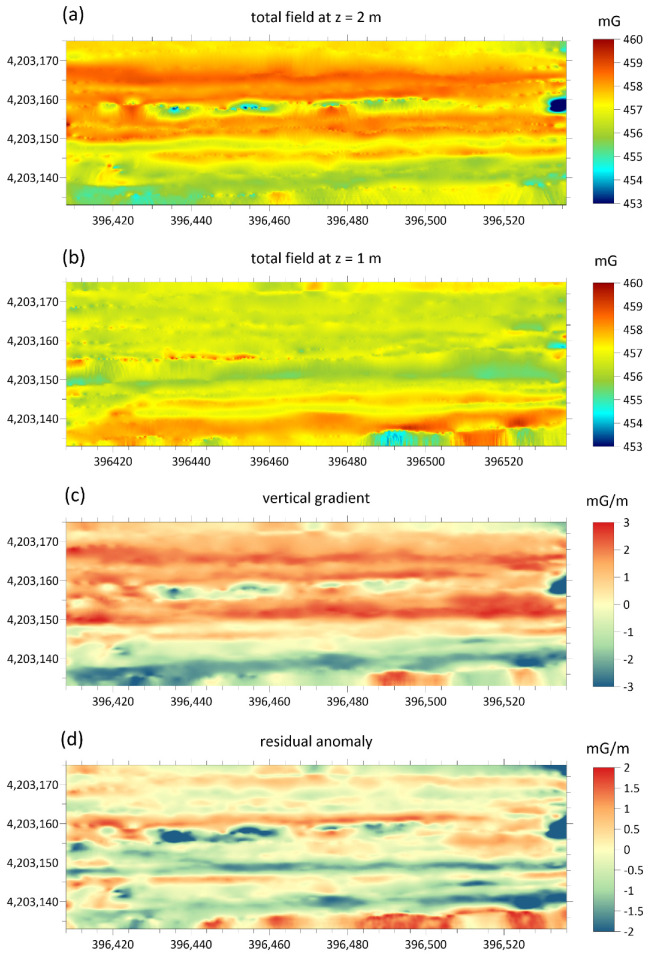
Steps for the calculations of the residual gradiometric anomaly related to the area covered by the first test. (**a**,**b**) Maps of the total magnetic field surveyed with the sensor at 3 and 2 m above ground level, respectively; (**c**) gradiometric maps resulting from the differences between the two maps; (**d**) residual anomaly after removing the background magnetic signal. Projected coordinates referred to UTM zone 33. Units of the magnetic field are in milligauss.

**Figure 8:**
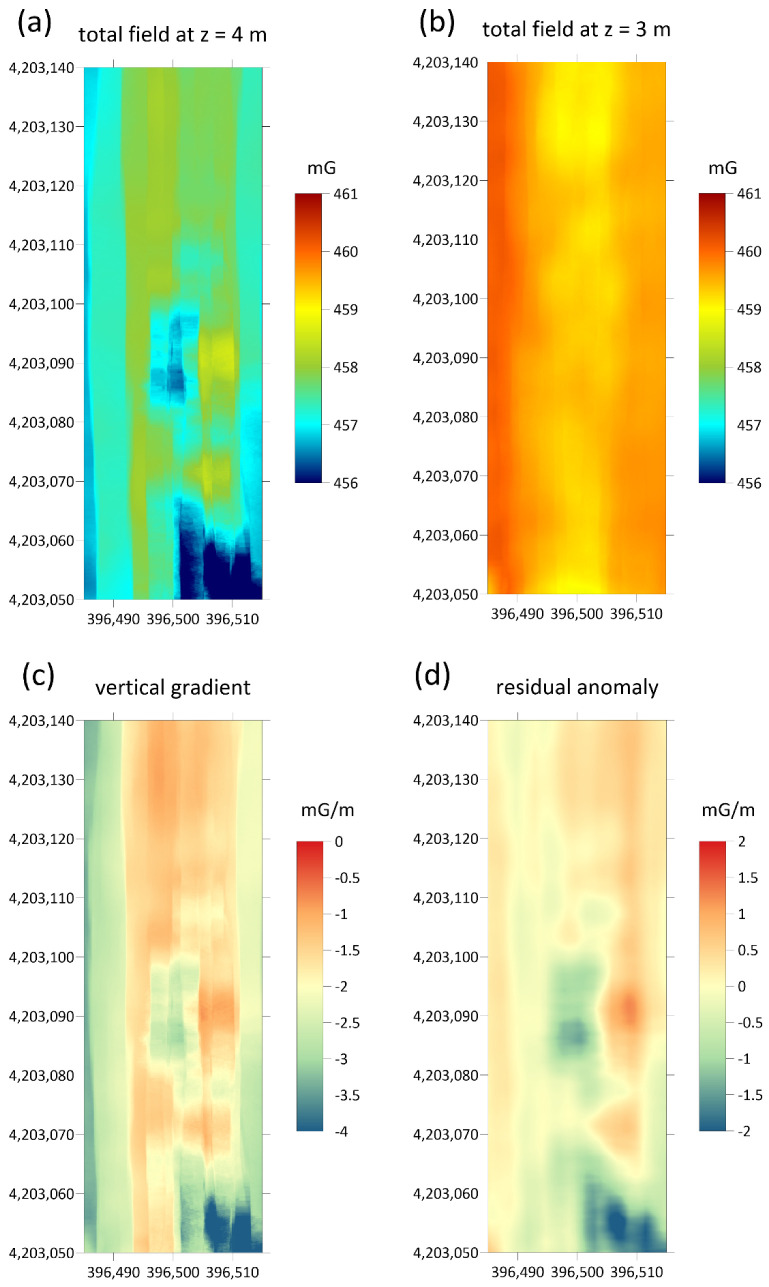
Steps for the calculations of the residual gradiometric anomaly related to the area covered by the second test. (**a**,**b**) Maps of the total magnetic field surveyed with the sensor at 4 and 3 m above ground level, respectively; (**c**) gradiometric maps resulting from the differences between the two maps; (**d**) residual anomaly after removing the background magnetic signal. Projected coordinates referred to UTM zone 33. Units of the magnetic field are in milligauss.

For each investigated area two maps of the total magnetic field were obtained whose overflight altitude differs by about one meter. These are shown in Figure 7a,b and Figure 8a,b. From them, by difference, the vertical gradient maps were obtained, in order to better highlight the superficial archaeological structures with respect to deeper anomalies or background. Furthermore, the vertical gradient data were filtered with a moving average with a window equal to 2 m along the flight direction and 0.5 m in the perpendicular direction, in order to eliminate the oscillations due to high-frequency noise in the measurements. The gradient maps thus obtained are shown in Figure 7c and Figure 8c. Finally, a background removal filter has been applied to the gradient maps, along the direction perpendicular to that of flight, in order to remove the local trend, probably due to altitude estimation errors, accidentally introduced by the imperfect maintenance of the UAV altitude and consequently to local variations in the difference in altitude between the two flights. The obtained residual anomaly maps are shown in Figure 7d and Figure 8d.

## 4. Discussion

The device presented in this work integrated a 3C-fluxgate magnetometer with a UAV platform, capable of performing magnetic surveys for a broad range of applications. The operational performances were tested on the field, selecting among the possible applications, one of the extreme cases, such as archaeological prospection where anomalies are usually subtle. For this test in particular, the system presented some unavoidable limitations, which were somehow compensated by the other advantages of the UAV magnetometry. In archaeological magnetic prospections, the sensor must be located as close to the ground as possible (some tens of cm), and the distance for gradiometry should be conveniently short. The UAV system allowed a minimum distance of the sensor from the ground of about 2 m and the distance for gradiometry equal to 1 m. Obviously, this arrangement was not optimized for the detection of superficial archaeological structures of small dimensions, such as wall structures which are typically a few tens of centimeters in size. However, this system should be able to highlight archaeological features even at greater depths and highlight the main structures, such as ancient roads whose dimensions reach a few meters. Thus, although we suffered from low resolution of archaeological features, we were nevertheless able to recognize the main alignments marked by anomalies with amplitudes < 1.0 mG (Figure 7d).

**Figure 9:**
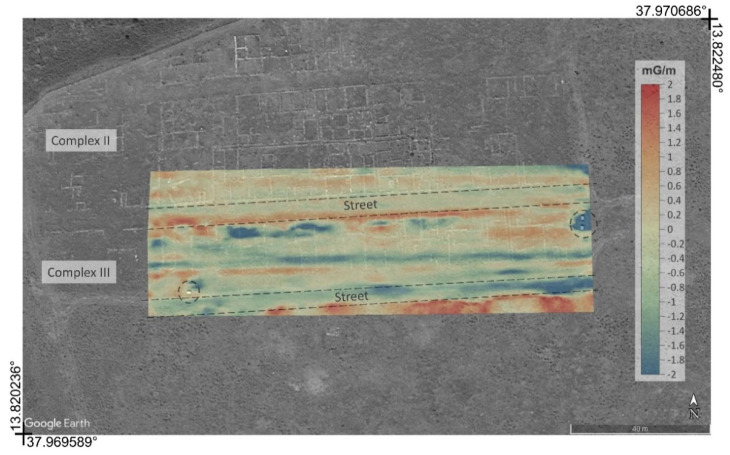
Overlay of the map of the residual magnetic anomaly with an aerial Google Earth image, the circle dots indicate the tourist information board. Units in milligauss/m.

The map of the residual anomaly showed a pattern which is in agreement with the archaeological features (Figure 9). Although it was not possible from the map of residual anomalies to accurately detect the archaeological structures present in the area, anomalies attributable to buried anthropogenic structures are highlighted. Both positive and negative anomalies are stretched along the ~E-W direction that corresponds to the main wall foundations beside the 6 m wide roads (Figure 2). Secondary anomalies also mark some of the minor wall foundations in ~N-S direction (i.e., complex II in Figure 2). Anomalies with a similar arrangement are also recognizable in the southernmost stripe of the surveyed area, where the site has not been excavated yet. Finally, in the easternmost portion of the site, the clear anomaly was due to a pair of metal boards also visible in the aerial photograph (Figure 9).

**Figure 10:**
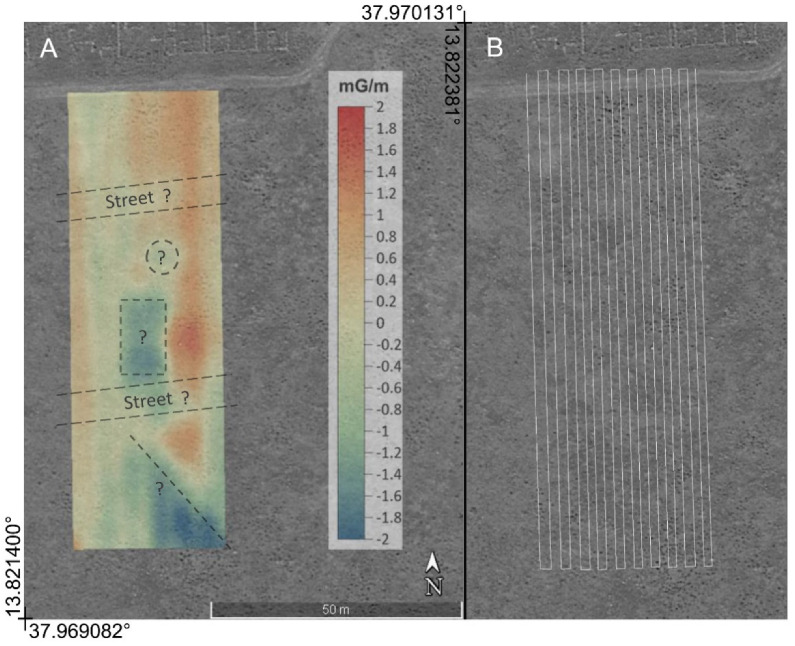
(**A**) Overlay of the map of the residual magnetic anomaly with an aerial Google Earth image. The line, circle, and square dots indicate supposed buried archaeological ruins. (**B**) The path of the performed mission follows N-S oriented tracks spaced 2 m. Units in milligauss/m.

The second map of the residual anomaly reported in Figure 10 shows a pattern indicating the presence of buried structures. This area is still unexplored, but it is supposed to be an extension of the ancient urban plant with a regular grid pattern. In fact, several geometrically well-defined anomalies were identified, most likely caused by anthropogenic structures. Some of them were elongated along the ESE-WNW direction, coinciding with that of the already identified streets in the urban plant, so a continuation of the latter could be hypothesized towards the south. Other clearly visible anomalies were well delimited and took on a rectangular or circular shape. They could be attributed to foundations of houses or factories with a morphology similar to those emerging in the northern sector, already excavated.

The advantage of the UAV magnetometry relies on the high density of measures, which avoid missing short-wavelength anomalies, and on the time-efficiency of the survey. In fact, large areas can be surveyed in a very short time if compared with the traditional survey method. The surveyed areas were about 5800 and 6700 m^2^ respectively, and they both were surveyed twice (at two different elevations) in a net time of about 23 min for each flight and gross time of 190 min, which included the device mounting, settings, and repeated battery changes, collecting >29,400 single measures over the two investigated areas.

The ability to survey large areas that require in-depth and detailed investigations makes the UAV magnetometry of great potential in archaeological sites. Although the system does not allow a high degree of resolution in the restitution of the buried archaeological structures, it must however be emphasized that the ability to identify and map the archaeological structures also depends on the contrast of the anomaly they produce. For this reason, some sites are easier to investigate than others.

Furthermore, considering the results of the system test on an archaeological site, it can reasonably be assumed that the system was more suitable for fields of application where the expected anomalies were of even greater intensity and/or greater scale. For example, mining exploration, the detection of buried metal objects or even the mapping of the magnetic structure of the subsoil are possible fields of application.

The UAV system was not a non-magnetic platform; several modules and engines were made of metal materials, which could have a significant influence on the magnetometers mounted to the UAV and reduce system measurement accuracy. The interference magnetic field from the UAV was one of the most significant hurdles to gathering high-quality magnetic data by the UAV-magnetometer system [28,37]. To mitigate the impacts of the UAV on the sensor, a long cable (4 m) was suspended below the UAV platform. This technical solution triggered oscillatory phenomena which could be conveniently filtered. Applying narrow stop-band filters, we considered as highly unlikely that the filtering might completely remove a real magnetic anomaly: it could perhaps be reduced in amplitude. In addition, the sensitivity of the fluxgate sensor to its attitude should be considered. This effect can be reduced while planning the survey; however, the results indicate that the spatial interpolation and the following processing were able to correct this bias.

## 5. Conclusions

The experimental set-up made it possible to achieve the set objectives of cost and effort reduction of any magnetic survey for the various fields of application. Tests carried out in a well-known archaeological site highlighted the system’s capability to detect low-amplitude anomalies caused by the most important archaeological structures. The whole system proved to be robust and reliable; however, some problems have been encountered related both to the rolling of the sensor, due to the wind and to the length of the cable, and to the non-perfect holding of the altitude established by the UAV. These issues caused systematic noises on the recorded signal which were partially attenuated during the data processing phase at the expense of decreased resolution. In the future, the oscillation of the sensor could be attenuated using a more rigid cable. In addition, the length of the cable can be conveniently varied when expected anomalies are of the same wavelength of the oscillations. However, being careful to avoid electromagnetic interference from the UAV’s motors. In addition, the integration of a more sensitive pressure gauge helped to have a precise and timely estimate of the UAV altitude, allowing us to perform a datum level correction of the data. Finally, a future software update would allow a real-time filtering of the signal and the preliminary magnetic map to be created in real time, during the survey.

Other similar systems which integrate magnetometers and UAV platforms have been proposed in the last years. Most of them are based on heavy multicopter, fixed-wing UAVs, or even prototypical UAVs, but the interference magnetic field from the UAV is one of the most significant limits to gathering high-quality magnetic data.

One of the strong points of this system is that it exploits an off-the-shelf, cheap, UAV platform, maybe the most popular one worldwide. Our device was lightweight, flexible, low interference, and reliable, and could be easily adopted by several professionals in many different applications. With the current configuration only large objects can be recognized; however, this configuration may already be useful for many archaeological purposes. Surely it can be extremely useful for carrying out a quick and low-cost first survey, preparatory to the execution of detailed surveys. The preventive execution of expeditious surveys with the system here implemented can lead to great advantages in terms of optimization of detailed investigations with possible significant savings in time and costs. Another aspect to take into consideration is that such investigations with UAVs can be carried out easily even in inaccessible sites. The last but not least advantage is the possibility of carrying out large scale expeditious surveys for geological purposes. As is well known, archaeology and geology are closely related disciplines, and it is often impossible to carry out a correct archaeological interpretation without having sufficient elements of knowledge on the local geology. Magnetic surveys can reveal anomalies attributable to underground geological bodies, cavities, landslides, or discontinuity surfaces attributable to faults, which can provide fundamental information for a correct archaeological interpretation [12].

We are working on a new upgraded survey platform that will provide a rigid magnetometer mount with two magnetometer sensors with a vertical alignment and new datalogger lightweighter then before. In this way, it will not be necessary to perform two flights at different elevation to measure the vertical gradient.

## Data Availability

The data that support the findings of this study are available from the corresponding author upon reasonable request.
